# All-in-one CRISPR/Cas-engineered glucocorticoid-receptor knock-out EBV-gp350-CAR knock-in T cells are potent and resistant to dexamethasone

**DOI:** 10.1186/s40164-025-00631-w

**Published:** 2025-03-19

**Authors:** Theresa Kaeuferle, Maximilian Zwermann, Nadine Stoll, Paulina Ferrada-Ernst, Lena Jablonowski, Reinhard Zeidler, Semjon Willier, Dana Stenger, Abdallah Yassin, Renata Stripecke, Tobias Feuchtinger

**Affiliations:** 1 Department of Pediatric Hematology, Oncology, Hemostaseology and Stem Cell Transplantation, Dr. von Hauner Children’s Hospital, University Hospital LMU Munich, Munich, Germany; 2 Center for Cell and Gene Therapy Freiburg, University Medical Center Freiburg, Albert-Ludwigs-University Freiburg, Freiburg, Germany; 3 Department of Pediatric Hematology and Oncology, University Medical Center Freiburg, Albert-Ludwigs-University Freiburg, Freiburg, Germany; 4 German Center for Infection Research (DZIF), Munich, Germany; 5 Department of Otorhinolaryngology, University Hospital LMU Munich, Munich, Germany; 6 Institute of Structural Biology, Helmholtz Munich, Neuherberg, Germany; 7 Institute of Translational Immuno-Oncology, Cancer Research Center Cologne-Essen (CCCE), Faculty of Medicine and University Hospital Cologne, University of Cologne, Cologne, Germany; 8 Faculty of Medicine and University Hospital Cologne, Department I of Internal Medicine Cancer Center Cologne Essen, Center for Integrated Oncology Aachen Bonn Cologne Düsseldorf, Center for Molecular Medicine Cologne (CMMC), University Hospital of Cologne, Cologne, Germany; 9 German Center for Infection Research (DZIF), Partner Site Hannover-Braunschweig, Hannover, Germany; 10 German Center for Infection Research (DZIF), Partner Site Bonn-Cologne, Cologne, Germany; 11 Clinic of Hematology, Hemostasis, Oncology and Stem Cell Transplantation, Hannover Medical School (MHH), Hannover, Germany; 12 German Cancer Consortium (DKTK), Partner Site Freiburg, Freiburg, Germany; 13 LETSimmun CRC TRR338 (SFB), Munich-Würzburg, Germany

**Keywords:** CAR T cells, EBV, CRISPR/Cas, Genetic engineering, Adoptive T cell transfer, Dexamethasone, Steroid treatment, Glucocorticoid receptor

## Abstract

**Background:**

Epstein-Barr virus (EBV) reactivation in immunocompromised patients and *post*-transplantation is associated with morbidity, mortality and with the onset of a variety of malignant diseases. Adoptive T-cell therapies have emerged as promising therapeutic options, but *post*-transplant immunosuppression jeopardizes the protective anti-EBV immune surveillance by adoptively transferred T cells.

**Methods:**

Using an all-in-one CRISPR/Cas-mediated approach, we inserted an anti-EBV (gp350) CAR into the T-cell receptor (*TRAC*) locus and simultaneously knocked-out the glucocorticoid receptor (GR) on a good manufacturing practice (GMP)-compatible platform.

**Results:**

CAR knock-in (CAR^KI^) was confirmed in primary human T cells on genetic and on protein level with a mean efficiency of 41%. With 83%, additional GR knock-out was highly efficient in CAR^KI^ cells. On a functional level CAR^KI^GR^KO^ T cells showed target-specific potency in terms of cytokine secretion patterns, proliferative capacity and cytotoxic activity against gp350-expressing target cells. Further, CAR^KI^GR^KO^ T cells were insensitive to dexamethasone treatment and maintained T-cell functionality. In contrast, CAR^KI^GR^KO^ T cells were sensitive to the GR-independent immunosuppressant cyclosporine A (CsA), thereby providing a rescue treatment for patients in case of safety issues.

**Conclusions:**

The study lays the proof-of-concept for virus-free all-in-one GMP-manufacturing of glucocorticoid-resistant CAR T-cell products. Further, the glucocorticoid-resistant gp350-CAR T cells can provide a future therapeutic option for high-risk *post*-transplant patients with EBV-reactivations or patients with EBV-associated pathologies requiring steroid treatment.

**Supplementary Information:**

The online version contains supplementary material available at 10.1186/s40164-025-00631-w.

## Background

Reactivations of the Epstein-Barr virus (EBV) can occur in immunocompromised hosts and patients after transplantation and are associated with morbidity, mortality and with potential malignant transformation leading to a variety of malignant diseases. Since T cells play a major role in the control of EBV re-activation, adoptive T-cell therapies have emerged as promising therapeutic options for EBV-associated lymphoproliferative diseases (LPD) or malignancies, especially in immunodeficient or immunosuppressed patients [1, 2]. EBV antigens, such as latent membrane proteins (LMP1 and LMP2) and nuclear proteins (EBNA1, -2, EBNA3A, -B, -C, and EBNA-LP) have been considered useful targets for the expansion of virus-specific T cells for adoptive T cell therapies [3, 4]. However, adoptive transfer of donor-derived EBV-specific T cells relies on the presentation of the antigenic peptide epitopes by the human leukocyte antigens (HLAs), which are highly polymorphic and downregulated in EBV-infected cells [5]. The EBV envelope glycoprotein gp350 (encoded by *BLLF1*) is expressed on the surface of infected cells during lytic reactivation and chimeric antigen receptor (CAR) T cells targeting gp350 (gp350-CAR T cells) showed HLA-independent cytotoxicity against EBV-induced lymphoproliferative diseases (LPD) and tumor development in humanized and xenograft mouse models [6–8]. Recently, gp350-CAR T cells received approval as an Innovative New Drug (IND) by the Center for Drug Evaluation (CDE) of China’s National Medical Products Administration (NMPA) and from the Unites States Food and Drug Administration (FDA) as an autologous T cell therapy for relapsed/metastatic nasopharyngeal carcinoma (NPC), with orphan drug designation. A phase I clinical trial completed recruitment in Q1/2024 to treat patients with NPC in China (ClinicalTrials.gov Identifier: NCT05864924).

*Post*-transplant patients under immunosuppressive therapy are at high risk of EBV reactivations and EBV-associated oncogenesis [9, 10]. Glucocorticoids potently exert immunosuppressive effects and remain the favored therapeutic option in a variety of clinical indications. However, besides the detrimental immunosuppression and abrogated immune surveillance against EBV, glucocorticoids also directly induce EBV lytic replication by upregulating BZLF1 gene expression [11, 12]. A variety of EBV-associated diseases outside the *post*-transplant cohort require steroid treatment, like EBV-triggered hemophagocytic lymphohistiocytosis [13], EBV^+^ NK/T cell lymphoma [14] or nasopharyngeal carcinoma. This provides a conflicting situation, in which immunosuppressive treatment in the high-risk patient population jeopardizes the protective anti-EBV immune surveillance by adoptive T-cell therapy. Glucocorticoids have been reported to suppress functionality of transferred T cells in terms of activation and expansion [15–17].

Clustered regularly interspaced short palindromic repeats/Cas (CRISPR/Cas) technology has revolutionized the field of genetic engineering due to its site-specificity and high efficiency [18]. The ability of CRISPR/Cas technology to facilitate efficient multiplex genome editing allows knock-in and knock-out in an all-in-one approach and therefore opens a new era of adoptive T-cell therapy products [19]. First clinical trials with multiplex CRISPR/Cas-engineered T cells revealed encouraging results in terms of safety, engraftment and feasibility [20, 21]. We have previously reported our strategy to render primary human virus-specific T cells glucocorticoid-resistant by CRISPR/Cas-mediated knock-out of the glucocorticoid receptor (GR) [22]. Characterization of the GR knock-out (GR^KO^) T cells demonstrated preserved protective T cell functionality parameters, such as cytotoxicity, CD107a degranulation, proliferative capacity, and cytokine release patterns [22].

Here, we used an all-in-one CRISPR/Cas-mediated GMP-compatible approach to insert a gp350-CAR into the T-cell receptor (*TRAC*) locus and simultaneously knock-out the glucocorticoid receptor. The novel gp350-CAR GR^KO^ T cells maintained strong target-specific potency also upon high-dose glucocorticoid treatment. Thereby we established a proof-of-concept manufacturing approach on a GMP-compatible platform to pave the way for clinical application of glucocorticoid-resistant CAR T-cell products.

## Methods

### Primary cells and cell lines

PBMCs were obtained from healthy donors after informed consent. Ethics approval was obtained from the local Research Ethics Committee (Approval number 36–16) and the study was performed in accordance with the Declaration of Helsinki. PBMCs were isolated from EDTA-blood by standard density gradient centrifugation.

The HEK-293T cell line (abbreviated HEK WT) was obtained from the American Tissue Culture Collection (CRL-11268TM 145 ATCC, ATCC, Manassas, Virginia, USA) and the HEK gp350 cell line was generated after lentiviral transduction (kindly provided by Prof. Renata Stripecke (Institute of Translational Immuno-Oncology, Cancer Research Center Cologne-Essen (CCCE), Faculty of Medicine and University Hospital Cologne, University of Cologne, Cologne DE) as previously described [6, 8]. Gp350 expression on HEK-293-T cells was re-confirmed by staining with a monoclonal 7A1 anti-gp350 Alexa647 antibody (kindly provided by Prof. Reinhard Zeidler, LMU Munich, Germany, Helmholtz Munich) 1:150 for 10 min at room temperature.

HEK WT-GFP and HEK gp350-GFP were generated after transduction with a retroviral vector carrying AcGFP1. In short, the packaging cell line HEK Vec-Galv was transfected with the plasmid pMP71-AcGFP1 for the transient production of viral particles, subsequently HEK293 Vec RD114 were transduced to generate a stable producer cell line. HEK WT and HEK gp350 were seeded at 1 × 10^6^ cells/ml and cultured overnight in DMEM supplemented with 10% FCS, 1% penicillin/streptomycin and 1% l-glutamin. Culture medium was removed and filtered virus supernatant was added to the cells to allow transduction for 48 h. Cells were expanded and FACS-sorted for GFP^+^ cells.

Daudi (ACC-78, German Collection of Microorganisms an Cell Cultures DSMZ, Brunswick, Germany) and Jiyoye (ACC-128, German Collection of Microorganisms an Cell Cultures DSMZ) lymphoma cell lines expressing fLuc-GFP were kindly provided by Prof. Renata Stripecke (Institute of Translational Immuno-Oncology, Cancer Research Center Cologne-Essen (CCCE), Faculty of Medicine and University Hospital Cologne, University of Cologne, Cologne DE).

### CRISPR/Cas genetic engineering

Research knock-out protocol: T cells were isolated from PBMCs using untouched magnetic enrichment via EasySep™ according to manufacturer’s instructions (Stemcell Technologies Inc., Vancouver, Canada). T cells were activated for 48 h using TransAct (anti-CD3/anti-CD28; Miltenyi Biotec, Bergisch-Gladbach, Germany) in TexMACS™ medium (Miltenyi Biotec) supplemented with 2.5% human AB serum and each 12.5 ng/ml IL-7 and IL-15 (both Miltenyi Biotec). crRNA-*TRAC* (GAGAAUCAAAAUCGGUGAAU for T-cell receptor engineering) or crRNA-*NR3C1* (CUUUAAGUCUGUUUCCCCCG for glucocorticoid receptor engineering) was complexed with tracrRNA (all Integrated DNA Technologies, Inc., Skokie, USA) by heat annealing at 95 °C. For the Ribonucleoprotein complex (RNP) Alt-R® S.p. Cas9 Nuclease V3 and Alt-R Cas9 Electroporation Enhancer (both Integrated DNA Technologies, Inc., Skokie, USA) were added to the complexed crRNA:tracrRNA in a 0.6:1 (Cas9:gRNA) ratio according to manufacturer’s instructions. For electroporation, T cells were resuspended in home-made Buffer M as previously published [23] and mixed with the RNP. Electroporation was performed with the Nucleofector Transfection 2b (Lonza, Basel, Switzerland) using T-023 program. After electroporation the T cells were in vitro expanded in TexMACS Medium plus 2.5% human AB serum supplemented with 12.5 ng/ml IL-7 and IL-15. MOCK control cells were electroporated without addition of RNPs.

Pre-GMP knock-out/knock-in protocol: Activated T cells were mixed with RNP complex containing the respective guideRNAs sgRNA-*TRAC* GAGAAUCAAAAUCGGUGAAU and sgRNA-*NR3C1* CUUUAAGUCUGUUUCCCCCG (both Synthego, Redwood City, California, USA) and sNLS-SPCAS9-sNLS enzyme (Aldevron, Fargo, North Dacota, USA) in a 1:0.3 ratio and Alt-R Cas9 Electroporation Enhancer (Integrated DNA Technologies, Inc.). For HDR samples, 8 µg HDR template (sequence provided by Prof. Renata Stripecke [8], synthesized by GenScript Biotech Corporation, Piscataway, New, Jersey, USA) was added. Cells were electroporated on the ExPERT GTx in Electroporation Buffer EP (both MaxCyte Inc., Rockville, Maryland, USA) via Expanded T-cell 4. After electroporation, HDR Enhancer V2 (Integrated DNA Technologies, Inc.) was added according to manufacturer’s instructions for 24 h. Cells were cultured in TexMACS™ medium supplemented with 2.5% hAB serum. After 30 min, IL-7 and IL-15 were added for a final concentration of 12.5 ng/ml. MOCK control cells were electroporated without addition of RNPs and HDR template.

### Evaluation of CAR knock-in efficiency

To detect the CAR knock-in on genetic level, DNA was isolated at day 7 *post* electroporation using the QIAamp DNA Mini Kit and PCR was performed using forward primer 5ʹ-CCCAACTTAATGCCAACATACCA-3ʹ, reverse primer 5ʹ-CAGGCCAGGTCCAGATTCTT-3ʹ and Q5® High-Fidelity DNA Polymerase (NEB M0491L) with the cycling parameters 98 °C for 30 s—33 cycles of 98 °C for 10 s, 66 °C for 30 s, 72 °C for 120 s—and 72 °C for 120 s—4 °C forever. PCR products were visualized in a 2% agarose gel with GelRed™ Nucleic Acid Gel Stain, 10,000X (b-41003 Biotium) in the Molecular Imager® Gel Doc™ XR System from Bio-RAD.

For evaluation of CAR knock- in efficiency on protein level, cells were in a first step stained 1:100 with FITC-labelled AffiniPure F(ab')₂ Fragment Goat Anti-Human IgG (Jackson ImmunoResearch Europe Ltd, Ely, UK), mixed and incubated 30 min on ice. Cells were subsequently washed and extracellularly stained with 7AAD (Biolegend, San Diego, California, USA), CD45-BV510 (Biolegend), CD4-APC-Cy7 (Biolegend), CD8-APC (BD), and TCR apha/beta PE-Cy7 (Biolegend) for 30 min on ice. 7AAD staining additionally allows for analysis of viability. Samples were washed with PBS before analysis with MACSQuant Analyzer 10 flow cytometer (Miltenyi Biotec) and FlowJo_V10.9.0 (FlowJo LLC, BD, Franklin Lakes, New Jersey, USA).

### Western blot

3 × 10^5^ cells were disrupted RIPA buffer for preparation of protein lysates and supplemented with 6 × Laemmli were separated and subsequently blotted onto a PVDF or nitrocellulose membrane. Membrane was blocked and stained with anti-GR antibody 1:500–1:1000 and anti-GAPDH antibody 1:2000 (both Santa Cruz Biotechnology Inc., Dallas, USA). Proteins were immunodetected with secondary goat anti-mouse IgG HRP antibody 1:5000 (Invitrogen, Waltham, Massachusetts, USA) and Clarity Western ECL Substrate (BioRad, Hercules, USA) or SuperSignal West Femto (Thermo Fisher Scientific Corp., Waltham, Massachusetts, USA). Blots were quantified using an ImageJ-based [24] digital image analysis approach.

### Immunosuppressive treatment

Dexamethasone (Sigma-Aldrich®, Merck KG) was dissolved in 10% DMSO at a stock concentration of 10 mM. Dexamethasone was added at 50 µM, 100 µM or 200 µM to cell culture medium as indicated. CsA (Thermo Fisher Scientific, Corp.) was dissolved in 100% DMSO at a stock concentration of 50 µg/ml. CsA was added to the culture medium 100 ng/ml, 400 ng/ml or 800 ng/ml as indicated. For negative controls without immunosuppression, DMSO was used and is abbreviated “w/o Dexa” or “w/o CsA”.

### Intracellular cytokine and activation marker staining

Expanded T cells were co-cultured with Daudi, Jiyoye, HEK WT or HEK gp350 in a 1:1 ratio (CAR T cells:target cells) in TexMACS™ (Miltenyi Biotec) supplemented with 2.5% human AB serum. After 1 day of co-culture 10 µg/ml Brefeldin A (Sigma-Aldrich®, Merck KG) and, where indicated, CD107a antibody (Miltenyi Biotec) was added for 4 h. For pre-gating on CAR-positive cells, cells were in a first step stained 1:100 with FITC-labelled AffiniPure F(ab')₂ Fragment Goat Anti-Human IgG (Jackson ImmunoResearch Europe Ltd, Ely, UK), mixed and incubated 30 min on ice. For analysis of cytokines and activation markers, cells were subsequently washed and stained for surface markers with CD4-VioGreen (Miltenyi Biotec) and CD8-APC-Cy7 (Biolegend) followed by intracellular staining with IFNγ-PE (Miltenyi Biotec), TNFα-PE-Cy7 (Biolegend) and CD154-VioBlue (Miltenyi Biotec) using the Inside Stain Kit according to the manufacturer’s instructions (Miltenyi Biotec). Samples were analyzed via MACSQuant Analyzer 10 flow cytometer (Miltenyi Biotec) and FlowJo_V10.9.0 (FlowJo LLC, BD).

### Proliferation

16 days *post* electroporation, T cells were labelled with 1 µM CTV for 5 min at 37 °C using the CellTrace™ Violet Proliferation Kit (Thermo Fisher Scientific Corp., Waltham, Massachusetts, USA) according to manufacturer’s instructions. After removal of unbound dye and washing according to manufacturer´s instructions, T cells were, where indicated, co-cultured with HEK WT or HEK gp350 target cells in a 1:1 ratio (CAR T cells: target cells) for 5 days in TexMACS™ medium (Miltenyi Biotec). Medium was changed after 3 days and dexamethasone was re-added respectively where indicated. Cells were extracellularly stained with FITC-labelled AffiniPure F(ab')₂ Fragment Goat Anti-Human IgG, CD4-APC-Cy7 (Biolegend) and CD8-APC-Cy7 (BD) as described above.

For assessing expansion rates *post* electroporation, total viable cells were microscopically counted after trypan blue staining at day of electroporation, day 2 or day 3 and day 5 *post* electroporation.

### Cytotoxicity assay

16 days after electroporation, T cells were co-cultured with HEK gp350 GFP in different effector: target ratios as indicated (CAR T cells: target cells) in TexMACS™ GMP medium (Miltenyi Biotec) for 5 days. HEK gp350 GFP without T cells served as control. IncuCyte® (Sartorius AG, Göttingen, Germany) imaging system was used to track GFP-positive target cells. GFP signal was documented every 3 h. Integrated Intensity was used for analysis.

For cytotoxicity assays with Daudi cells, Daudi target cells were labelled using the CellTrace™ Violet Proliferation Kit (Thermo Fisher Scientific Corp., Waltham, Massachusetts, USA) according to manufacturer’s instructions. CAR^KI^ cells and Daudi cells were co-cultured in TexMACS GMP medium (Miltenyi Biotec) at an E: T ratio of 0.5: 1 for 48 h. For calculation of cytotoxic capacity, living CellTrace™ Violet-positive target cell count in co-cultured wells was divided by the mean of living CellTrace™ Violet-positive target cell count in control wells (target cells only). Ratios are depicted in % cell lysis. Co-culture of target cells with MOCK T cells served as control.

### Cytokine release in supernatant

T cells were co-cultured with HEK WT or HEK gp350 in a 1:1 ratio (CAR T cells:target cells) in TexMACS™ GMP Medium (Miltenyi Biotec). After 2 days of co-culture supernatants were harvested and frozen at − 20 °C until analysis. Secreted cytokines were analyzed with the bead-based immunoassay LegendPlex (Biolegend) according to manufacturer’s instructions.

### Statistics

Graphs were generated and data were statistically analyzed via GraphPad Prism_V9.1.0 (Graphpad Software Inc., Boston, Massachusetts, USA). Paired student’s *t*-test was used unless otherwise noted. * p < 0.05, ** p < 0.01, *** p < 0.001, *** p < 0.001 **** p < 0.0001 were considered significant.

## Results

### All-in-one CRISPR/Cas-mediated generation of gp350-CAR^KI^TCR^KO^GR^KO^ T cells

**Fig. 1:**
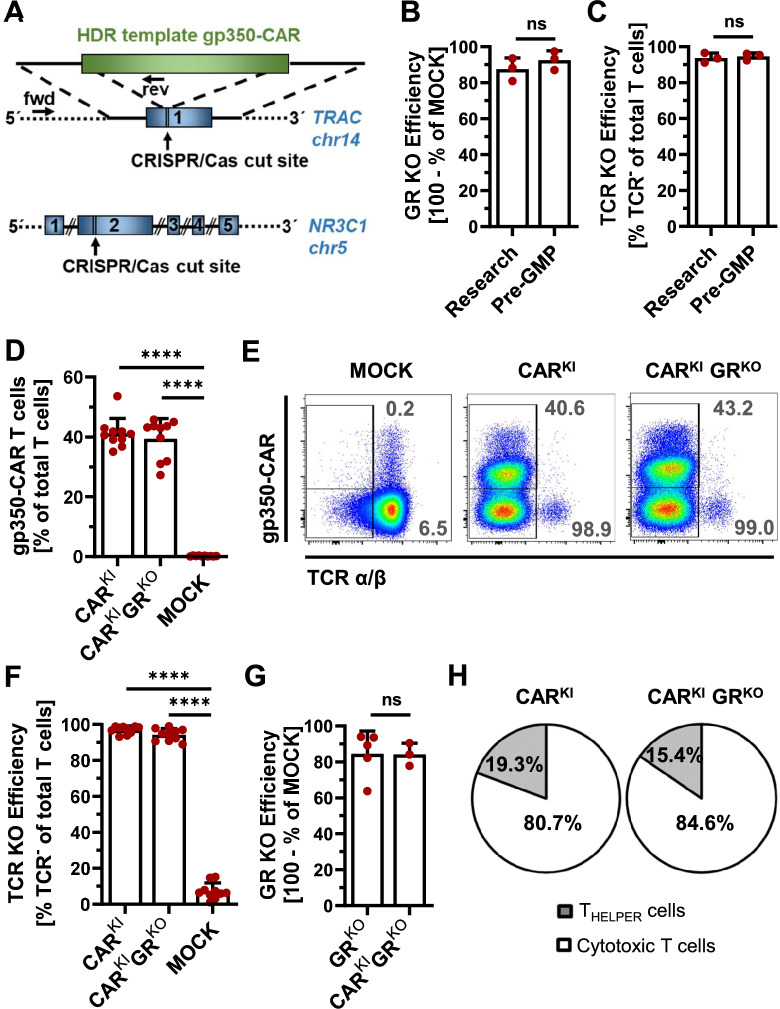
CRISPR/Cas-mediated generation of glucocorticoid resistant gp350-CAR T cells. **A** Scheme of CRISPR/Cas genetic engineering approach and location of primer pairs used for confirmatory PCR. Gp350-CAR was inserted into exon 1 of T-cell receptor alpha constant chain (*TRAC*) gene via homology-directed repair (HDR) with simultaneous glucocorticoid receptor (GR) knock-out via double-strand break in *NR3C1* exon 2. **B** GR knock-out efficiency on protein level generated by research and pre-GMP protocol determined by ImageJ-based analysis of Western Blot. Mean + SD of n = 3 donors. **C** TCR knock-out efficiency on protein level generated by research and pre-GMP protocol determined by flow-cytometric staining of TCRα/β. Mean + SD of n = 3 donors. **D** gp350-CAR knock-in efficiency on protein level determined by flow-cytometry. Mean + SD (n = 10 donors). **E** Exemplary flow cytometry density plots of CAR knock-in cell staining in MOCK electroporation control, GR wildtype (CAR^KI^) and GR knock-out (CAR^KI^GR^KO^) samples. **F** TCR knock-out efficiency on protein level determined by flow-cytometry. Mean + SD (n = 10 donors). **G** GR knock-out efficiency on protein level in TCR wildtype T cells and in sorted CAR^KI^ T cells evaluated via Western Blot and ImageJ-based digital image analysis. Protein levels were normalized to those of GAPDH. Mean + SD (n = 3 and 5 donors as indicated). Unpaired student’s *t*-test. **H** Flow-cytometric detection of T_HELPER_ vs. cytotoxic T-cell ratios via CD4 single positive and CD8 single positive T-cell populations. Mean of n = 10 donors. **** p < 0.0001; ns = not significant

In order to generate glucocorticoid-resistant multiply edited CAR T cells targeting the EBV envelope glycoprotein gp350, the anti-gp350 CAR was inserted into the T-cell receptor alpha chain (*TRAC*) locus via CRISPR/Cas-mediated homology-directed repair, thereby knocking-out the T-cell receptor. Simultaneously the *NR3C1* gene encoding for the glucocorticoid receptor (GR) was knocked-out in an all-in-one approach (Fig. 1A).

Firstly, we upgraded the gene knock-out approach from a research protocol using research reagents and a research device towards a GMP-compatible approach using pre-GMP Cas/gRNA reagents (Aldevron/Synthego), pre-GMP electroporation buffer and GMP device (pre-GMP protocol). GR knock-out was highly efficient with 88.4% ablation of *NR3C1* gene and 92.6% knock-out of GR expression using the pre-GMP protocol (Fig. 1B, Supplemental Fig. 1A, C). We did not observe significant differences in editing efficiencies between the research and the pre-GMP protocol, neither on DNA level (83.1% research vs. 88.4% pre-GMP, Supplemental Fig. 1A) nor on protein level (87.6% research vs. 92.6% pre-GMP, Fig. 1B, Supplemental Fig. 1C). In accordance, the T-cell receptor (TCR) knock-out efficiency was not significantly different between the protocols, neither on *TRAC* DNA level (86.0% research vs. 86.7% pre-GMP; Supplemental Fig. 1D) nor on TCR protein level (93.8% research vs. 94.7% pre-GMP; Fig. 1C, Supplemental Fig. 1F). Additional comparative analyses in terms of cell viability as well as expansion rates *post* electroporation confirmed the results. Cell viabilities were high and did not significantly differ between research and pre-GMP protocols at day 13 *post* electroporation, neither for GR KO samples (83.4% research vs. 87.4% pre-GMP, Supplemental Fig. 1B) nor for TCR KO samples (96.6% research vs. 95.4% pre-GMP, Supplemental Fig. 1E). In terms of early expansion *post* electroporation, viable cell counts increased 4.4-fold in the research TCR KO sample and 3.8-fold in the pre-GMP TCR KO sample within the first 5 days (Supplemental Fig. 1G). In the GR KO sample, cells expanded 4.3-fold (research) and 3.7-fold (pre-GMP) until 5 days *post* electroporation (Supplemental Fig. 1G). Expansion rates were not significantly different between research and pre-GMP knock-out samples.

Secondly, efficiency of CAR knock-in via the pre-GMP protocol was assessed. Successful CAR knock-in in primary human T cells was confirmed on genetic level via PCR analyses of *TRAC* locus independent of additional GR knock-out (Supplemental Fig. 2A). No bystander CAR off-target integration in the GR locus was detectable by PCR (Supplemental Fig. 2A). Flow-cytometric determination of CAR and T-cell receptor expression on protein level showed a mean of 41.2% CAR knock-in efficiency with a concurrent TCR knock-out efficiency of 97.0% (Fig. 1D–F). Additional GR knock-out did not change CAR knock-in efficiency significantly (41.2 to 39.4%, p = 0.30) and slightly reduced the TCR knock-out efficiency (97.0 to 94.3%, p = 0.003) (Fig. 1D, F). Western Blot analysis showed GR knock-out efficiency of 84.6% in bulk T cells **(**Fig. 1G). For the detection of GR knock-out efficiencies in CAR T cells, CAR T cells were FACS-sorted prior to Western Blot. GR knock-out efficiencies were not significantly impaired by additional CAR knock-in (84.6% GR^KO^ vs. 83.1% CAR^KI^GR^KO^; p = 0.85, Fig. 1G).

Further we analyzed ratios between cytotoxic T cells (CD8 single positive CAR T cells) and T_HELPER_ cells (CD4 single positive CAR T cells). GR wildtype as well as the GR^KO^ CAR-T-cell populations mainly consisted of CD8^+^ gp350-CAR T cells (80.7% and 84.6%, respectively) with CD4^+^ gp350-CAR T cells in the minority (19.3 and 15.4%, respectively, Fig. 1H). Thus, frequencies of CD8^+^ gp350-CAR T cells were significantly higher than frequencies of CD4^+^ gp350-CAR T cells (p < 0.0001) in both GR wildtype and GR^KO^ cells.

### Functional characterization of GR knock-out CAR^KI^ cells compared to GR wild-type CAR^KI^ cells

**Fig. 2:**
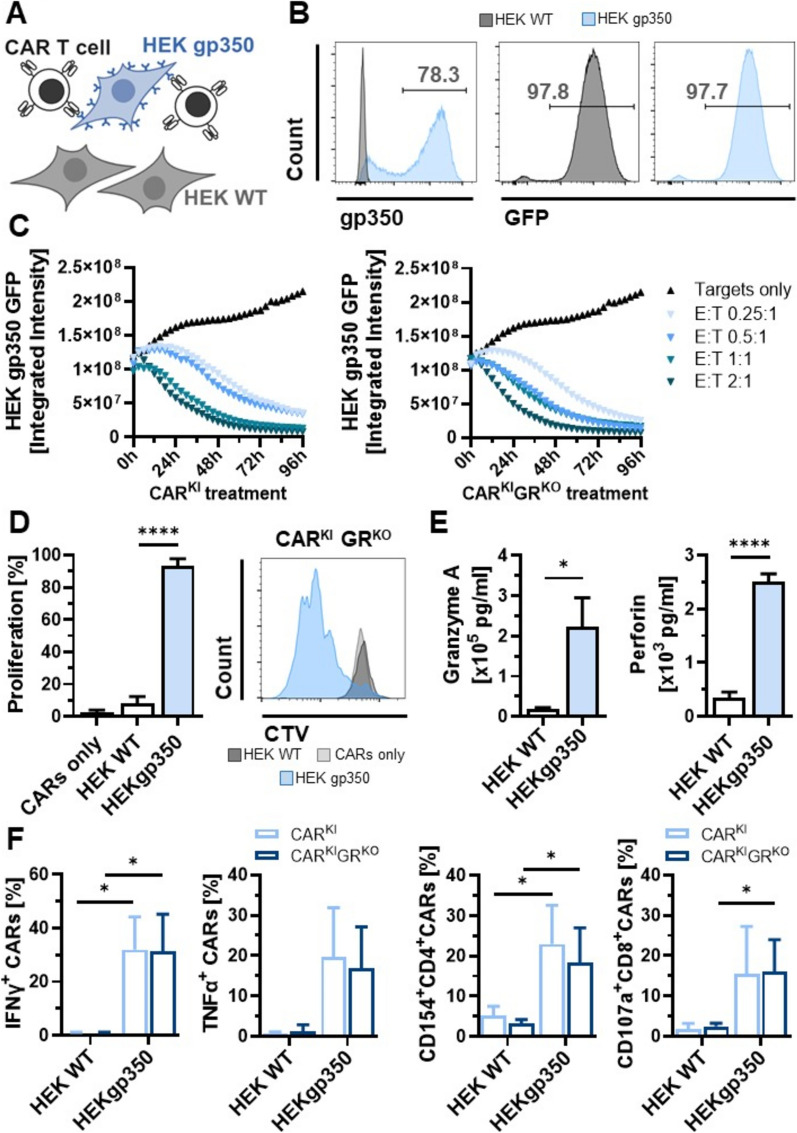
Functional characterization of GR knock-out CAR T cells compared to GR WT CAR T cells. **A** Scheme of gp350-transduced HEK293T cells (HEK gp350) and HEK293T wild-type (HEK WT) target cells for assessment of target-specific functionality of CAR T cells. Created with biorender. **B** Flow cytometry histograms of gp350 staining and GFP signal of HEK293T cell lines used for functional characterization. **C** IncuCyte’s GFP Integrated Intensity of gp350^+^ HEK293T cells (HEK gp350 GFP) upon treatment with CAR^KI^ or CAR^KI^GR^KO^ cells. Mean of 2 donors and different effector:target (E: T) ratios as indicated. Gp350^+^ HEK293T GFP cells without T cells were used as control (Targets only). **D** Proliferation capacity as determined via CellTrace™ Violet (CTV) staining of CAR^KI^GR^KO^ cells upon HEK WT or HEK gp350 co-cultures. CAR^KI^GR^KO^ cells without addition of target cells were used as control (CARs only). Mean + SD (n = 4 donors). **E** Exemplary graphs from Fig. 3A showing absolute concentrations of Granzyme A and Perforin levels detected in the supernatants of co-cultures via multiplex assay. Mean + SD (n = 4 donors, each performed in technical duplicates). **F** Frequencies of IFNγ^+^ and TNFα^+^ among CAR T cells, CD154^+^ cells among CD4^+^CAR T cells and CD107a^+^ cells among CD8^+^CAR T cells upon co-culture with HEK WT or HEK gp350 target cells. T cells were intracellularly stained and pre-gated on CAR^+^ T cells in CAR^KI^ or CAR^KI^GR^KO^ samples as indicated. Mean + SD (n = 4 donors). * p < 0.05, **** p < 0.0001

In order to assess the effects of the GR knock-out in gp350-CAR T cells (CAR^KI^GR^KO^), the functionality of CAR^KI^GR^KO^ cells was assessed by cytotoxicity, cell proliferation and cytokine secretion and compared to GR wild-type CAR T cells (CAR^KI^). Target-specificity was investigated upon co-culture of the CAR T cells with HEK293T expressing gp350 (HEK gp350) compared to wild-type cells (HEK WT) (Fig. 2A, B).

For comparing functionality of CAR^KI^ and CAR^KI^GR^KO^ T cells in terms of cytotoxicity potency, HEK gp350 target cells co-expressing GFP were used (Fig. 2B). Cytotoxic capacity against gp350-transduced target cells was confirmed and dependent on the effector-to-target ratio (E: T) (Fig. 2C). In contrast, WT target cells were not killed by CAR^KI^ nor by CAR^KI^GR^KO^ T cells, thereby confirming target-specific cytotoxic activities (Supplemental Fig. 2C). GR knock-out did not impair the target-specific cytotoxic capacity of the CAR^KI^ T cells (Fig. 2C).

In addition, co-culture of CAR^KI^GR^KO^ cells with HEK gp350 led to higher proliferative capacity of CAR^KI^GR^KO^ (93.5%) compared to CAR^KI^GR^KO^ cells only (2.6%) or in co-culture with gp350-negative cells (HEK WT) (8.2%, Fig. 2D).

Analysis of cell culture supernatants revealed high up-regulation of cytotoxic markers granzyme A (from 0.2 to 2.2 × 10^5^ pg/ml) and perforin (from 0.3 to 2.5 × 10^3^ pg/ml, Fig. 2E) upon stimulation of CAR^KI^GR^KO^ cells with HEK gp350 target cells. The ability of the CAR^KI^GR^KO^ cells to release cytokines target-specifically was additionally shown via intracellular staining of the T_h1_-cytokines IFNγ and TNFα as well as activation markers. Gp350 stimulation strongly up-regulated the cytokines IFNγ (from 0.8 to 31.9% of CAR T cells) and TNFα (from 0.75 to 19.6% of CAR T cells; Fig. 2F) as well as the T_HELPER_ cell marker CD154 (from 5.1 to 23.0% of CAR T_HELPER_ cells) and cytotoxic T-cell marker CD107a expression (from 1.8 to 15.4% of cytotoxic CAR T cells; Fig. 2F).

**Fig. 3:**
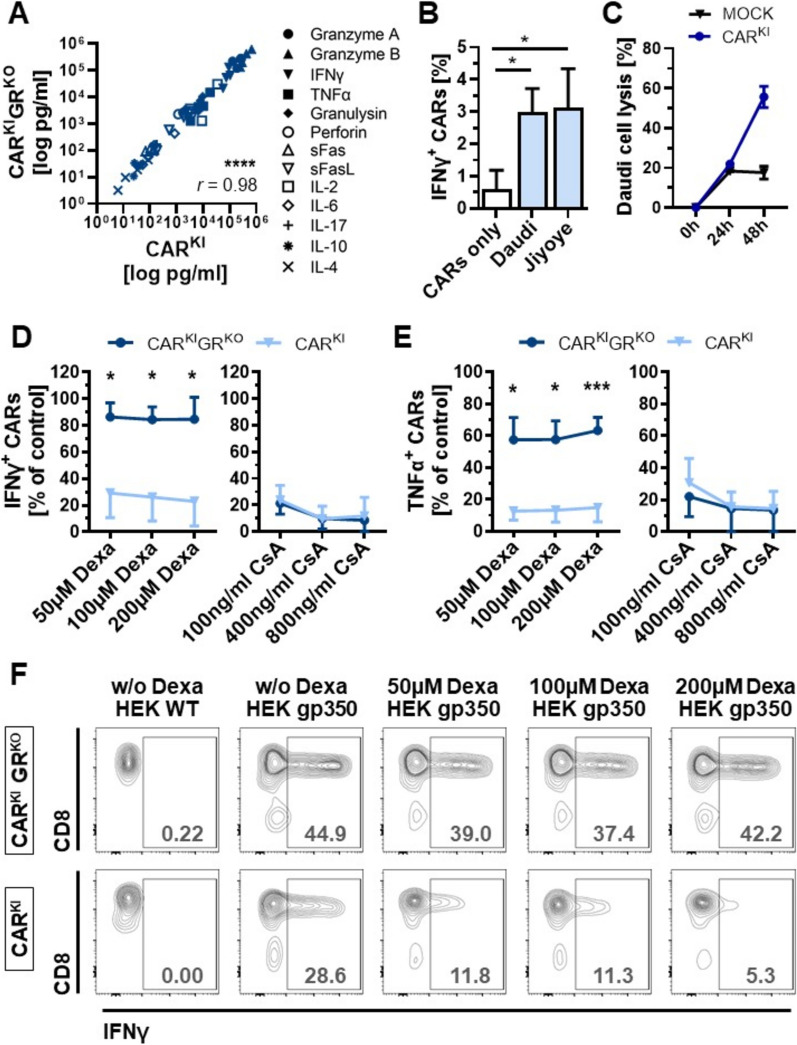
GR knock-out rescues cytokine secretion upon glucocorticoid treatment. **A** Correlation of absolute cytokine concentrations determined via multiplex analysis in cell culture supernatants of wild-type GR CAR T cells (CAR^KI^) or CAR T cells with additional GR knock-out (CAR^KI^GR^KO^) co-cultured with HEK gp350. n = 4 donors; each data point presents the mean of technical duplicates from 1 donor. Pearson correlation; *r* = Pearson r. **B** Frequencies of IFNγ^+^ cells among CAR T cells upon co-culture with Daudi or Jiyoye cells. Mean + SD of n = 3 donors. **C** Cytotoxic capacity of CAR^KI^ cells on Daudi cells (E: T ratio 0.5: 1) upon 0 h, 24 h and 48 h of co-culture. Daudi co-culture with MOCK T cells served as control. Mean ± SD of 1 donor performed in technical duplicates. **D** Percent of CAR^+^IFNγ^+^ T-cell frequencies in dexamethasone (Dexa) or cyclosporine A (CsA)-treated samples among CAR^+^IFNγ^+^ T-cell frequencies in the respective control cells without any immunosuppressant. Mean ± SD (n = 3 donors Dexa, n = 6 donors CsA). Asterisks indicate significant differences between CAR^KI^ and CAR^KI^GR^KO^ samples. **E** Percent of CAR^+^TNFα^+^ T-cell frequencies in dexamethasone (Dexa) or cyclosporine A (CsA)-treated samples among CAR^+^TNFα^+^ T-cell frequencies in the respective control cells without any immunosuppressant. Mean ± SD (n = 3 donors Dexa, n = 6 donors CsA). Asterisks indicate significant differences between CAR^KI^ and CAR^KI^GR^KO^ samples. **F** Representative flow cytometry contour plots of IFNγ staining. Cells were pre-gated on CAR^+^ T cells. * p < 0.05, *** p < 0.0001

In order to investigate the influence of the additional GR knock-out on cytokine secretion, up-regulation of cytokines was compared between CAR^KI^GR^KO^ cells and GR wild-type CAR T cells (CAR^KI^) cells. Up-regulation was independent of additional GR knock-out for IFNγ (31.9 vs. 31.2%, p = 0.88), TNFα (19.6 vs. 16.8%, p = 0.22), CD154 (23.0 vs. 18.3.6%, p = 0.19) and CD107a expression (15.4 vs. 16.0%, p = 0.90; Fig. 2F). Within the analysis of cell culture supernatants, a pattern of T_h1_ as well as T_h2_-cytokines and markers (granzyme A, granzyme B, IFNγ, TNFα, granulysin, perforin, sFas, sFasL, IL-2, IL-6, IL-17, IL-10 and IL-4) showed highly significant correlation between the CAR^KI^ and CAR^KI^GR^KO^ cells upon HEK gp350 stimulation (r = 0.98, p < 0.0001, Fig. 3A). Stimulation with gp350-transduced target cells led to a significant increase in absolute cytokine levels in the supernatant, exemplary shown for granzyme A and perforin levels in Fig. 2E. Highest absolute concentrations upon co-culture with HEK gp350 were detected for the cytoplasmic granule proteases granzyme A and granzyme B and the T_h1_ cytokine IFNγ (Fig. 3A).

In order to investigate the targeted efficacy of the CAR T cells on EBV-driven malignant cells, we co-cultured the CAR^KI^ cells with Daudi and Jiyoye B lymphoblast cell lines isolated from Burkitt’s Lymphoma patients. 3% of Daudi and 1% of Jiyoye cells expressed gp350 in our in vitro setting (Supplemental Fig. 2B). However, significant up-regulation of IFNγ-secretion upon co-culture with Daudi (fivefold) or Jiyoye cell lines (5.2-fold) confirmed the ability to detect these target cells in vitro (Fig. 3B). Accordingly, Fig. 3C shows the cytotoxic capacity of CAR^KI^ cells on Daudi cells (56% target cell lysis after 48 h of co-culture).

### GR knock-out rescues CAR T-cell activity upon glucocorticoid dexamethasone treatment

The effect of GR knock-out on CAR T-cell functions upon treatment with the glucocorticoid dexamethasone was investigated via staining of CAR^KI^ cells for detection of cytokines and activation marker levels. In control samples without addition of an immunosuppressant,

Upon treatment with increasing concentrations of dexamethasone, IFNγ up-regulation was strongly impaired in GR wild-type cells to 0.29, 0.26 and 0.23 of the expression detected in the control cells without dexamethasone treatment respectively (Fig. 3D, F). GR knock-out rescued IFNγ expression significantly back to 0.84—0.86 independent of dexamethasone concentration (Fig. 3D, F). Also TNFα expression was highly affected by dexamethasone treatment in GR wild-type cells (0.13–0.15 of control without dexamethasone treatment), whereas GR knock-out significantly rescued TNFα expression back to 0.57–0.63 even upon high-dose dexamethasone treatment (Fig. 3E, Supplemental Fig. 2D).

**Fig. 4:**
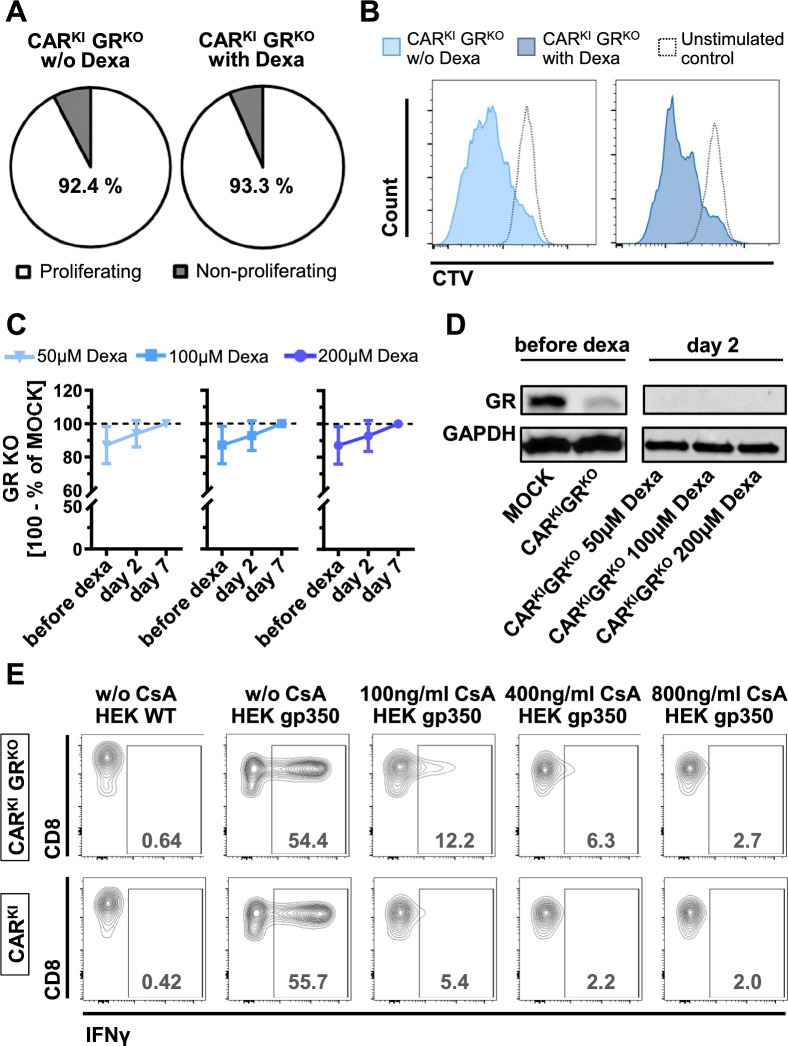
Effects of immunosuppressive treatment on CAR^KI^GR^KO^ cells. **A** Proliferation capacity as determined via CellTrace™ Violet (CTV) staining of CAR^KI^GR^KO^ cells upon HEK WT (unstimulated control) or HEK gp350 co-cultures. CAR^KI^GR^KO^ and HEK gp350 were co-cultured without any immunosuppressant (w/o Dexa, n = 4) and with 100 µM dexamethasone (n = 3). **B** Exemplary flow cytometry histograms of CTV staining. **C** GR knock-out efficiency on protein level before, at day 2 and day 7 of 50/100/200 µM dexamethasone (Dexa) treatment. Protein levels were evaluated via Western Blot and ImageJ-based digital image analysis and were normalized to those of GAPDH. Mean ± SD (n = 3 donors). Unpaired student’s *t*-test. **D** Exemplary Western Blot pictures of GR and GAPDH in MOCK and CAR^KI^GR^KO^ T-cell samples as indicated. **E** Representative flow cytometry contour plots of intracellular IFNγ staining upon 100/400/800 ng/ml CsA treatment. Cells were pre-gated on CAR^+^ T cells

Accordingly, dexamethasone treatment did not influence proliferation capacity in CAR^KI^GR^KO^ T cells (93.3% vs. 92.4%, p = 0.8, Fig. 4A, B).

### Dexamethasone-mediated enrichment of GR^KO^ cells

Moreover, dexamethasone treatment further enriched GR knock-out cells. Low-level dexamethasone treatment of 50 µM already increased the frequency of GR knock-out from 87.1% to 94.0% after 2 days of dexamethasone treatment (Fig. 4C). Similarly, both, treatment with 100 µM and 200 µM dexamethasone increased GR knock-out frequency to 92.8% after 2 days of dexamethasone treatment (Fig. 4C, D). 7 days of dexamethasone treatment completely eliminated GR wildtype cells independent of the dexamethasone concentration (Fig. 4C).

### GR-independent sensitivity of CAR^KI^GR^KO^ to immunosuppression

In order to ensure patient safety, we investigated the effect of a GR-independent immunosuppressive drug on glucocorticoid-resistant CAR T cells. Cyclosporine A (CsA) is a commonly used drug in the *post*-transplantation setting exerting its immunosuppressive effect GR-independently via inhibition of the phosphatase calcineurin. Treatment with low-dose CsA (100 ng/ml) already lead to suppressed IFNγ and TNFα secretion of 0.21 and 0.31 of control cells without CsA treatment (Figs. 3D, E, 4E). Increasing doses of CsA further decreased IFNγ and TNFα secretion to 0.09 and 0.14 of control cells upon 800 ng/ml treatment. Thus, in contrast to dexamethasone treatment, GR knock-out cells were sensitive to the suppressive effect of CsA in terms of IFNγ and TNFα secretion.

## Discussion

The results demonstrate efficient pre-GMP CRISPR/Cas-mediated gene editing in terms of an all-in-one approach including gp350-CAR knock-in, T-cell receptor knock-out and glucocorticoid receptor knock-out. The resulting T cells show strong target-specific potency combined with glucocorticoid resistance.

CAR T cells approved for clinical application are usually generated using retroviral and lentiviral vectors [18]. However, virus-free generation of CAR T cells by CRISPR/Cas genetic engineering shows several benefits, such as site-directed integration, defined regulation of the inserted gene product, financial efficacy due to modular systems and combinatorial multiple engineering options [18]. For clinical quality control of potential therapeutic cell products, flow-cytometric purity and impurity detection as well as potency assays and microbiological assessments can be adopted from virally transduced CAR T-cell products [18]. However, validation of cost- and time-effective approaches for detecting genotoxicity in terms of off-target effects and chromosomal rearrangements play a major role in translation of CRISPR/Cas-engineered cell products [18].

Clinical translation of CRISPR/Cas-engineered cells is still in its infancy and general safety issues remain to be investigated in prospective clinical studies. Pilot studies with CRISPR/Cas-engineered cells revealed encouraging results in terms of safety, engraftment and feasibility for sickle cell disease, ß-thalassemia or HIV [20, 21, 25]. The world’s first CRISPR/Cas-engineered cell therapy has been approved for sickle cell disease and ß-thalassemia and showed promising results not only in terms of safety but also in terms of efficacy [26].

T cells transduced with a chimeric antigen receptor (CAR) targeting the lytic EBV antigen gp350 are in clinical trials as adoptive immunotherapy against EBV-associated tumors (ClinicalTrials.gov Identifier: NCT05864924). Virus-free generation of gp350-CAR T cells using a CRISPR/Cas approach and evaluation of CAR functionality in Burkitt lymphoma mouse models has been published before [8]. In order to render the gp350-CAR T cells glucocorticoid-resistant for a high-risk *post*-transplantation setting, we not only extended the approach for virus-free combinatorial multiplex genetic engineering with additional glucocorticoid receptor (GR) knock-out but also upgraded the protocols towards GMP production using pre-GMP Cas/gRNA reagents (Aldevron, Synthego), pre-GMP electroporation buffer and the GMP-compatible electroporation device ExPERT GTx® (both MaxCyte). By using the GMP-compatible approach we roughly doubled published gp350-CAR knock-in efficiencies [8] with and without additional knock-out of the GR. In general, additional GR knock-out did neither impair knock-in efficiencies nor gp-350 CAR functionality.

It has been previously described, that dexamethasone treatment suppresses activation and effector function in T-cell responses [27]. Our data on gp350-CAR T cells confirm that genetic knock-out of the GR rescues these functions upon glucocorticoid treatment as published for virus-specific T cells in vitro [22, 28]. Menger et al. have additionally confirmed the efficiency of glucocorticoid receptor knock-out to render T-cells resistant to glucocorticoids in vivo [28]. Potential safety issues in terms of uncontrolled CAR T-cell activation in clinical application were addressed by evaluating sensitivity to GR-independent immunosuppressive drugs. In accordance with previously published data on virus-specific T cells [22], we confirm sensitivity of GR knock-out gp350-CAR T cells to cyclosporine A (CsA), an immunosuppressant acting via calcineurin inhibition. Thus, CsA treatment can provide a potential rescue treatment option for patients in case of uncontrolled CAR T-cell activation. However further in vivo evaluation of effectiveness and safety of the all-in-one gp350-CAR GR knock-out T-cell product is crucial for translation of the T-cell product towards clinical application.

In conclusion, the glucocorticoid-resistant gp350-CAR T-cell product provides a potential therapeutic option for *post*-transplant patients with EBV-reactivations or with EBV-associated pathologies upon glucocorticoid therapy. In addition, the presented approach lays the groundwork for GMP-compatible CRISPR/Cas-engineering to generate glucocorticoid-resistant CAR T-cell products for various clinical applications.

## Supplementary Information

Supplementary Material 1.

## Data Availability

No datasets were generated or analysed during the current study.

## Abbreviations

CAR: Chimeric antigen receptor
CRISPR/Cas: Clustered regularly interspaced short palindromic repeats/Cas
CsA: Cyclosporine A
EBV: Epstein-Barr virus
FACS: Fluorescence activated cell sorting
GR: Glucocorticoid receptor
gp350: Glycoprotein 350
GMP: Good Manufacturing Practice
HEK: Human embryonic kidney cells
HLAs: Human leukocyte antigens
IFNγ: Interferon γ
KI: Knock-in
KO: Knock-out
NPC: Nasopharyngeal carcinoma
PCR: Polymerase chain reaction
TCR: T-cell receptor
TRAC: T-cell receptor alpha chain
TNFα: Tumor necrosis factor α
WT: Wild-type
