# Supplementary material for: All-in-one CRISPR/Cas-engineered glucocorticoid-receptor knock-out EBV-gp350-CAR knock-in T cells are potent and resistant to dexamethasone
Source: Exp Hematol Oncol. 2025 Mar 19;14:40. doi: 10.1186/s40164-025-00631-w (PMC11921674; doi:10.1186/s40164-025-00631-w)
Supplement: Supplementary file 1 — Supplementary Material 1. [file 40164_2025_631_MOESM1_ESM.pdf]

Supplement
Figure S1

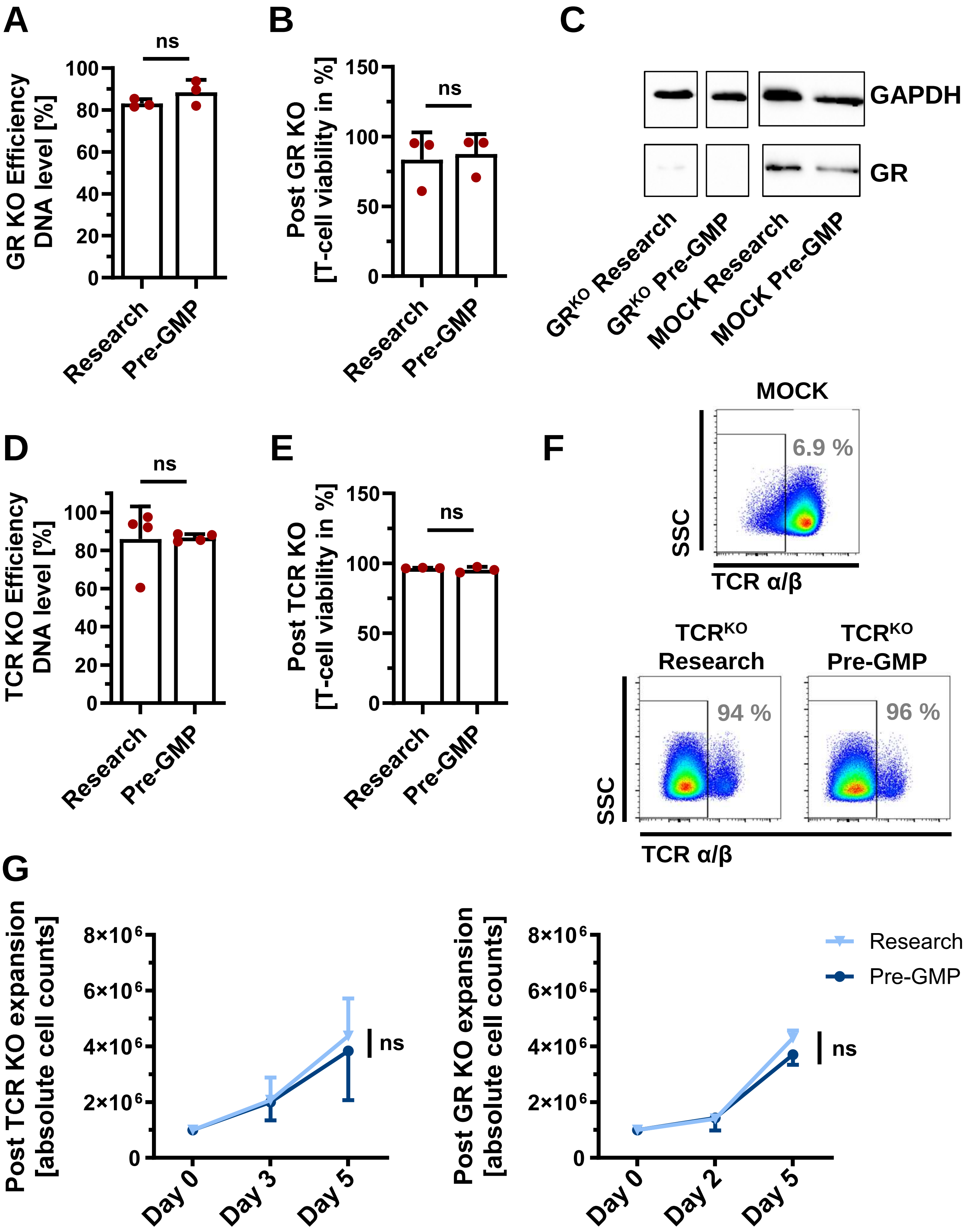

Figure S1 Pre-GMP Upscale of genetic engineering protocol.

A GR knock-out efficiency generated by research and pre-GMP protocol determined on DNA level by PCR amplification of *NR3C1* locus and TIDE analysis. Mean+SD of n=3 donors. **B** Cell viability of GR KO samples at day 13 *post* electroporation determined *via* flow cytometry after 7-AAD staining. Frequency of 7-AAD negative among T cells. Mean+SD of n=3 donors. **C** Exemplary Western Blot pictures of GR^{KO} and MOCK samples. **D** TCR knock-out efficiency generated by research and pre-GMP protocol determined on DNA level by PCR amplification of *TRAC* locus and TIDE analysis. Mean+SD of n=4 donors. **E** Cell viability of TCR KO samples at day 13 *post* electroporation determined *via* flow cytometry after 7-AAD staining. Frequency of 7-AAD negative among T cells. Mean+SD of n=3 donors. **F** Exemplary flow cytometry plots of GR^{KO} and MOCK samples. **G** Viable white blood cell absolute counts determined microscopically after trypan blue staining at day of electroporation (day 0), day 2 or day 3 and day 5 *post* electroporation for TCR KO and GR KO samples as indicated. Mean +/- SD of n=3 donors. ns=not significant

Supplement
Figure S2

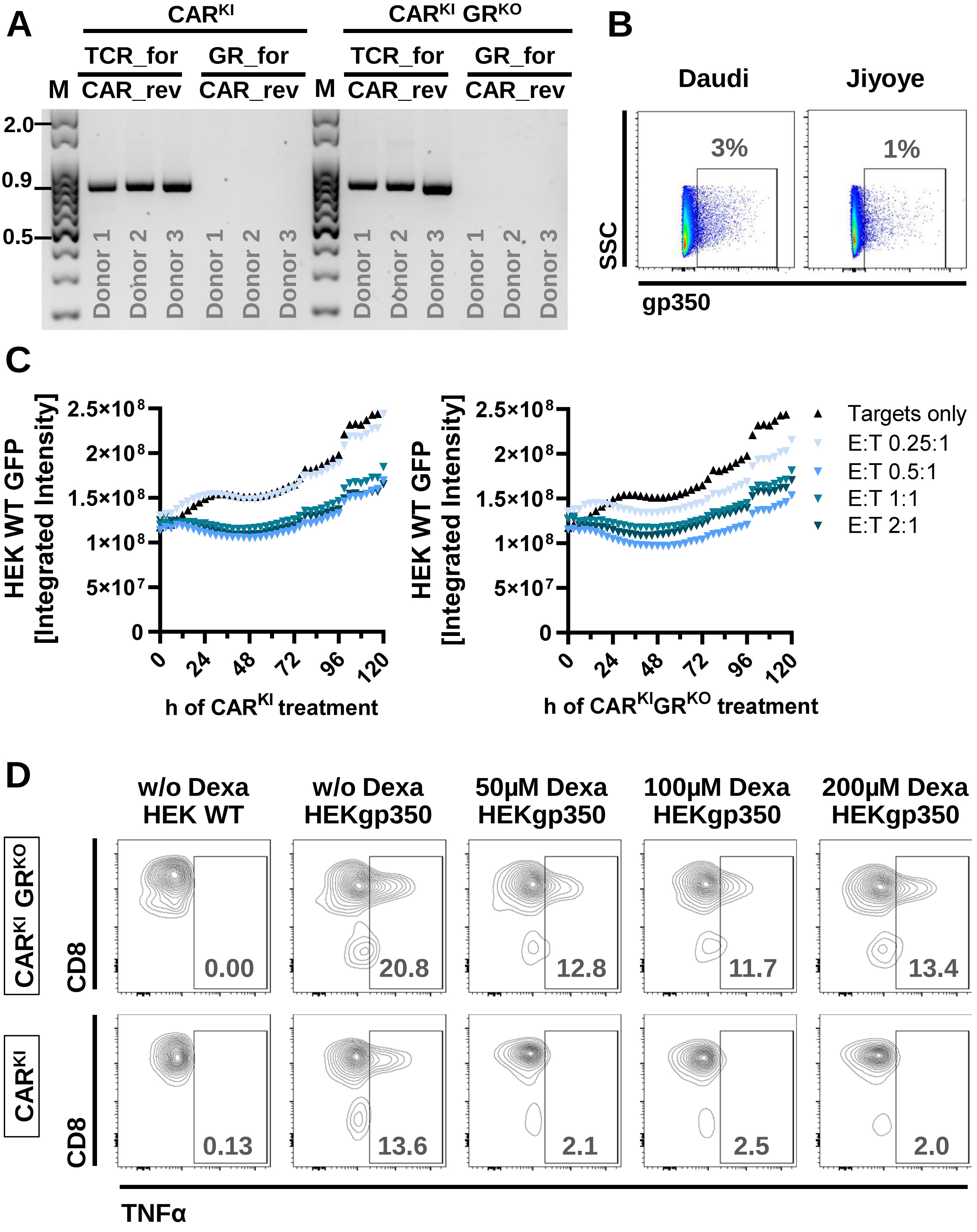

Figure S2 Genetic and functional confirmation of $CAR^{KI}GR^{KO}$ cells.

A Detection of CAR knock-in on genetic level. PCRs were performed with forward primer located in TRAC intron upstream HDR template’s homology arm binding site and reverse primer located in HDR template’s CAR coding region. PCR products‘ agarose gel picture of gp350-CAR knock-in cells with wildtype GR (CAR^{KI}) or with additional GR knock-out ($CAR^{KI}GR^{KO}$) from 3 donors. **B** Flow cytometric staining with anti-gp350 antibody for the detection of gp350 on target B-cell lines isolated from Burkitt‘s lymphoma patients. **C** IncuCyte’s GFP Integrated Intensity of HEK293T WT cells upon treatment with CAR^{KI} or $CAR^{KI}GR^{KO}$ cells in different effector:target (E:T) ratios as indicated. HEK293T WT cells without T cells were used as control (Targets only). **D** Percent of $TNF\alpha^{+}$ T-cell frequencies among total CAR T cells in dexamethasone (Dexa)-treated samples. Representative flow cytometry contoure plots of TNFα staining. Cells were pre-gated on CAR^{+} T cells.
